# ESM-Kredite zur Bekämpfung der Corona-Krise greifen zu kurz

**DOI:** 10.1007/s10273-020-2650-2

**Published:** 2020-05-20

**Authors:** Hans-Bernd Schäfer

**Affiliations:** Bucerius Law School Hamburg, Jungiusstraße 6, 20355 Hamburg, Deutschland

**Keywords:** K00, E52, E58

## Abstract

Am 9. April 2020 einigten sich die Finanzminister der Eurozone auf Kreditlinien beim Europäischen Stabilisierungsmechanismus (ESM), um der Corona-Krise entgegenzutreten. Die ESM-Kredite werden mit 200 Mrd. Euro beziffert. Dieser Wert ist jedoch irreführend, denn nur wenige Staaten hätten überhaupt Vorteile gegenüber einer zehnjährigen Staatsanleihe im eigenen Land. Zudem ist der Umfang der ESM-Kredite begrenzt.

## Abstract (en)

On 9 April 2020, the euro area finance ministers agreed on credit lines with the European Stability Mechanism (ESM) to counter the corona crisis. The ESM loans are estimated at 200 billion euros. However, this figure is misleading. Only a few states would benefit at all over a 10-year government bond in their own country. Moreover, the scope of ESM loans is limited.

Nach zweitägigem Verhandlungsmarathon über ein europäisches Finanzierungsprogramm zur Bewältigung der Corona-Krise einigten sich die Finanzminister der Eurozone am 9. April unter anderem auf Kreditlinien beim Europäischen Stabilisierungsmechanismus. Alle weitergehenden Vorschläge aus Frankreich, Italien und Spanien aber auch von vielen angesehenen Wirtschaftswissenschaftlern in Deutschland sind damit erst einmal vom Tisch. Gleichwohl ist es der Bundesregierung gelungen, dies als einen Durchbruch europäischer Solidarität darzustellen. Die Schlagzeilen der Weltpresse sprachen von einer Kreditlinie von 240 Mrd. So heißt es in euronews am 10. April 2020 „Governments can apply for access – under certain conditions – to a total of €240 billion being made available under the European Stability Mechanism“ (Sandford, 2020). Die Süddeutsche Zeitung titelte bereits am 25. März 2020 „240 Milliarden aus Rettungsschirm“ (Finke, 2020). Auf der Homepage der Bundesregierung (2020) heißt es „Die Eurogruppe hat sich auf ein Schutzprogramm über 500 Milliarden Euro geeinigt“. Damit werden indirekt die ESM-Kredite mit 200 Mrd. Euro beziffert. Dies sind weitverbreitete, aber irreführende Behauptungen.

## Nur fünf Staaten hätten Zinsvorteile von ESM-Krediten

Die Finanzminister der Eurozone schlugen vor, dass jeder Mitgliedstaat unter der in Art. 15 des ESM-Vertrags normierten Enhanced Conditioned Credit Line (ECCL) einen Betrag von 2 % seines Bruttoinlandsprodukts als Kredit aufnehmen kann, einen „Pandemic Crisis Support“ (Consilium Europa EU, 2020). Von den in Art. 3 und Art. 12 des ESM-Vertrags normierten strengen Auflagen wird abgesehen. Einzige Voraussetzung soll die Verwendung des Kredits für solche Kosten des Gesundheitswesens sein, die durch die Corona-Krise entstehen.

Doch wie attraktiv sind die Corona-Kredite für die Mitgliedstaaten der Eurozone? Der ESM refinanziert sich mit Krediten einschließlich Anleihen,[1] deren Bedienung die Mitgliedstaaten der Eurozone entsprechend ihrem Anteil am Eigenkapital garantieren. Die deutsche Quote beträgt 26,9 % (ESM, 2020). Entsprechend der Kreditwürdigkeit der einzelnen Mitgliedstaaten bilden sich für die Refinanzierungskredite des ESM Zinssätze heraus, die gleich dem mit den Eigenkapitalquoten gewichteten Durchschnitt aller Zinssätze sind, welche die Mitgliedstaaten auf den nationalen Anleihemärkten für ihre Schulden zahlen müssen. Dieser Zinssatz liegt zurzeit bei 0,53 %. Hinzu kommt eine Zinsspanne, welche die „volle Abdeckung“ aller Kosten des ESM ermöglicht (Art. 20 (1) ESM-Vertrag). Im April 2020 gibt der ESM seinen Ausleihzinssatz mit 0,76 % an. Es ist durchaus plausibel, dass für die Corona-Kredite die Zinsspanne etwas gesenkt wird, weil die milde Konditionalität dieser Kredite die Verwaltungskosten vergleichsweise niedrig hält. Zurzeit ist aber der Ausleihzinssatz von 0,76 % maßgeblich.

Mit diesem Zinssatz als Vergleich stellt sich heraus, dass 14 von den 19 Staaten der Eurozone für nationale Staats-

1 Die in der öffentlichen Diskussion vielfach geschmähten Eurobonds existieren somit bereits und sind in Art. 21 (1) ESM-Vertrag ausdrücklich normiert. „Der ESM ist befugt, zur Erfüllung seiner Aufgaben an den Kapitalmärkten bei Banken, Finanzinstituten oder sonstigen Personen und Institutionen Kapital aufzunehmen.“

**Prof. Dr. Hans-Bernd Schäfer** lehrt Law and Economics an der Bucerius Law School, der Hochschule für Rechtswissenschaft in Hamburg.

anleihen mit einer Laufzeit von zehn Jahren einen niedrigeren Zinssatz als für ESM-Kredite zahlen (vgl. Tabelle 1). In nur fünf Euroländern ist ein ESM-Kredit preiswerter als nationale Staatsanleihen. Das wussten selbstverständlich auch die Finanzminister der Eurostaaten, als sie am 9. April 2020 ihre Beschlüsse fassten. Gegenüber der europäischen Öffentlichkeit insinuierten sie aber, es existiere ein viel höheres Kreditvolumen. Sie bliesen einen Luftballon auf, der für einige Tage die gewünschten Schlagzeilen produzierte, der ESM würde 240 Mrd. Euro zur Bekämpfung der Corona-Krise bereitstellen.[2]

Werfen wir nun einen Blick auf jene Länder der Eurozone, für die ein ESM-Kredit preiswerter ist als nationale Verschuldung. Dies sind Griechenland, Italien Portugal, Spanien und Zypern. Die höchstmögliche Kreditsumme, die jene Länder überhaupt beantragen können, für die ein ESM-Kredit einen Zinsvorteil gegenüber der nationalen Verschuldung bewirkt, liegt insgesamt bei unter 70 Mrd. Euro und beträgt somit 29 % jener Summe, die von Politikern und Zeitungen herausposaunt wird. Gäbe es einen politischen Verbraucherschutz, so könnte jeder Bürger durch eine einstweilige Anordnung erwirken, dass die irreführende Information auf der Homepage des Finanzministeriums gelöscht würde. Für zwei der fünf Länder ist der finanzielle Vorteil so geringfügig, dass er sich fast verflüchtigt. Um diesen schätzen zu können, enthält Spalte 3 von Tabelle 2 die derzeitige Differenz zwischen dem Zinssatz für zehnjährige nationale Staatsanleihen und dem Zinssatz für ESM-Kredite. Multipliziert man diese Differenz mit dem höchstmöglichen ESM-Kredit jedes Landes, erhält man die höchstmögliche Zinsersparnis pro Jahr und Land. Nennenswerte Beträge – bezogen auf die Größe des Landes – können sich nur Italien, Griechenland und Zypern ausrechnen. Für Portugal und Spanien sind die Ersparnisse nicht der Rede wert und so niedrig, dass sich die Frage stellt, ob nicht die zusätzlichen Transaktionskosten eines ESM-Kredits wie Verhandlungs- und Berichterstattungskosten gegenüber dem ESM die Kreditaufnahme von vornherein unattraktiv machen.

Für die übrigen drei Länder ergeben sich bescheidene, aber nennenswerte Vorteile. Diese können aber über eines nicht hinwegtäuschen: Der ESM-Kreditrahmen ist – auch im Verbund mit weiteren Kreditlinien der Europäischen Kommission von 100 Mrd. Euro und der Europäischen Investitionsbank von 200 Mrd. Euro – so gering, dass diese Staaten ihre nationale Verschuldung erheblich ausweiten müssten, wenn es nicht noch zu einer anderen Lösung kommt.

Tabelle 1
**Zinssätze für zehnjährige Staatsanleihen in den Mitgliedstaaten der Eurozone**
in %

| Mitgliedstaat der Eurozone | Zinssatz, Stand: 13. April 2020 |
|---|---|
| Belgien | 0,087 |
| Deutschland | -0,349 |
| Estland | n.v. |
| Finnland | -0,240 |
| Frankreich | 0,100 |
| *Griechenland* | *1,776* |
| Irland | 0,206 |
| *Italien* | *1,587* |
| Lettland | -0,040 |
| Litauen | 0,310 |
| Luxemburg | -0,360 |
| Malta | 0,553 |
| Niederlande | -0,086 |
| Österreich | 0,087 |
| *Portugal* | *0,856* |
| Slowakei | 0,430 |
| Slowenien | 0,731 |
| *Spanien* | *0,777* |
| *Zypern* | *1,929* |
| Durchschnitt aller Staaten der Eurozone | 0,539 |

Quellen: Bloomberg, Eurostat und ESM, die Werte für Luxemburg, Finnland, Lettland und Litauen sind vom Februar 2020.

Die Zinsersparnis für die Länder, die überhaupt einen Vorteil davon haben, einen ESM-Kredit aufzunehmen, beträgt pro Jahr nach dieser Rechnung 347,8 Mio. Euro. Diese Ersparnis führt nicht zu laufenden Belastungen im Bundeshaushalt, sondern ist Folge der Staatsgarantie. Die deutsche Quote an dieser Garantie ist 26,9 %. Wenn alle fünf Länder mit relativ hohen Zinsen einen ESM-Kredit aufnehmen, beträgt die Gesamtkreditsumme 69,1 Mrd. Euro und der deutsche Haftungsanteil davon im schlimmsten Fall, wenn alle Kredite völlig ausfallen, 18,6 Mrd. Euro.

## ESM-Kredite, Konditionalität und Outright Monetary Transactions der Europäischen Zentralbank

Die Bundesregierung behauptet, der ESM sei ein etablierter, eingespielter Krisenmechanismus, der schnell ein-

2 Gegenwärtig ist unklar, wie langfristig die von den Finanzministern vorgeschlagenen Corona-Kredite überhaupt sein sollen. Nach einer internen Richtlinie des ESM hat der von den Finanzministern vorgesehene ECCL-Kredit nur eine Laufzeit von einem Jahr und kann zweimal für sechs Monate verlängert werden. Er ist eine Liquiditätshilfe und entspricht nicht den langfristigen Notwendigkeiten von Corona-Krediten. Dies kann zwar durch Beschluss der Finanzminister für die Corona-Kredite geändert werden. Offizielle Dokumente schweigen dazu, und diejenigen, die es wissen könnten, halten sich bei Nachfragen bedeckt.

Tabelle 2
**Mögliches Kreditvolumen und Ersparnis der fünf Staaten, für die ein ESM-Kredit vorteilhaft ist**

| | Eurozone, Bruttoinlandsprodukt 2019 zu Marktpreisen in Mrd. Euro | Maximaler ESM-Kredit zur Bekämpfung der Corona-Pandemie von 2 % des BIP in Mrd. Euro | Zinsersparnis des ESM-Kredits im Vergleich zu zehnjähriger nationaler Staatsanleihe in Prozentpunkten | Zinsersparnis pro Jahr in Mio. Euro |
|---|---|---|---|---|
| Alle Staaten der Eurozone | 11.905 | 238,10 | | |
| Griechenland | 187 | 3,74 | 1,016 | 38,0 |
| Italien | 1.788 | 35,76 | 0,827 | 295,7 |
| Portugal | 212 | 4,24 | 0,096 | 4,1 |
| Spanien | 1.245 | 24,90 | 0,017 | 4,9 |
| Zypern | 22 | 0,44 | 1,169 | 5,1 |
| Summe der fünf Länder | 3.454 | 69,08 | | |

Quelle: Eurostat, ESM, Bloomberg.

setzbar sei. Dies geht ziemlich leichtfüßig darüber hinweg, dass die Corona-Kredite des ESM mit dessen Statuten nicht übereinstimmen. Weder entsprechen sie den generellen Zielen des ESM, Mitgliedern mit schwerwiegenden Finanzierungsproblemen unter strikten Auflagen eine Stabilitätshilfe zu gewähren, noch ist die ECCL für den Zweck einer Seuchenbekämpfung eingerichtet worden. Was der ESM jetzt tut, ist mit einem Automobilkonzern vergleichbar, der beschließen würde, für eine Übergangszeit Schafzucht zu betreiben, statt Autos zu produzieren. Nun ist der ESM keine börsennotierte Gesellschaft, für die derartiges völlig undenkbar wäre, sondern eine juristische Person mit Sitz und Hauptverwaltung in Luxemburg. Er kam durch einen völkerrechtlichen Vertrag außerhalb des institutionellen Rahmens des Europarechts zustande. Letztinstanzlich würde der Europäische Gerichtshof (EuGH) nach Art. 37 III ESM-Vertrag mittels völkerrechtlicher Auslegung darüber entscheiden, ob diese Konstruktion ohne Satzungsänderungen erlaubt ist. Es existieren auch im deutschen Recht Ausnahmetatbestände, die es z. B. einer GmbH erlauben, durch Gesellschafterbeschluss von der Satzung abzuweichen, wenn die Entscheidung dringend ist und keine dauerhafte Änderung mit sich bringt (insbesondere Harbarth, 2018; und Zöllner und Noack, 2019). Die rechtlichen Probleme dieser aus Not und Zeitnot entstandenen Entscheidungen werden hier nur angedeutet.

Wesentlich erscheint aber, dass mit der Aufnahme eines ESM-Kredits zur Bekämpfung der Corona-Krise weitreichende Konsequenzen für das Gesamtgefüge des Europäischen Währungssystems verbunden sein können. Dies gilt vor allem für das Außerkraftsetzen der strikten Konditionalität und die Frage, wie sich dies auf die Zulässigkeit der Outright Monetary Transactions (OMT) der Europäischen Zentralbank (EZB) auswirkt. Die Bedingungen für einen Corona-Kredit zur Verbesserung des Gesundheitswesens müssen anders und wesentlich milder sein als für einen ESM-Kredit, welcher der Wiederherstellung finanzieller Stabilität dient. Es stellt sich die Frage, welche Auswirkungen die Aufnahme eines ESM-Kredits im Rahmen des „Pandemic Crisis Support" auf die Möglichkeit der Zentralbank hat, Staatsanleihen einzelner Länder aufzukaufen, wenn diese in eine Finanzkrise geraten.

Als auf dem Höhepunkt der Finanzkrise 2012 Italien unter den Druck der Finanzmärkte geriet und die dortigen Zinssätze für Staatsanleihen auf ein nicht tragfähiges Niveau anstiegen, hielt der Präsident der Europäischen Notenbank Mario Draghi seine berühmte Rede „Whatever it takes". Er kündigte ein Programm an, Staatsanleihen eines in Bedrängnis geratenen Mitgliedstaats aufzukaufen, vorausgesetzt dieser Staat habe vorher einen Kredit beim ESM aufgenommen, der strikter Konditionalität unterliegt. Allein diese Ankündigung ließ die Zinsen für italienische Staatsanleihen rasch sinken.

Dieser sogenannte Draghi-Plan war Gegenstand von Entscheidungen des Bundesverfassungsgerichts und des Europäische Gerichtshofs. Er wurde als zulässig akzeptiert, sowohl europarechtlich als auch vereinbar mit der deutschen Verfassung. Die Begründungen betrachten es aber als wesentlich, dass die OMT der Notenbank zur Stützung einzelner Mitgliedstaaten von den strikten Auflagen der Aufnahme eines ESM-Kredits begleitet sind. Die Europäische Zentralbank hatte in einer Presseerklärung vom September 2012 diese Bedingungen klar formuliert, an die sie OMT-Käufe knüpft (European Central Bank, 2012). Danach ist die „strikte und effektive Konditionalität" eines ESM-Kredits notwendige Voraussetzung für jegliche OMT-

Käufe, die zudem nach dieser Erklärung beendet werden, sobald der kreditnehmende Staat die Auflagen nicht erfüllt. Der EuGH stellte fest, dass dieses Programm vom Mandat der Zentralbank gedeckt ist und wies in seiner Begründung besonders auf die strikte Konditionalität hin.[3]

Wie sind nun OMT-Käufe der EZB von Staatsanleihen eines Landes zu beurteilen, das einen ESM-Kredit zur Deckung Corona-bedingter Kosten des Gesundheitswesens aufnimmt? Dessen Konditionen sind gänzlich anders als im Draghi-Plan vorgesehen. Reicht es für OMT-Käufe der Zentralbank aus, wenn ein auf eine Staatsinsolvenz zusteuerndes Land nachweist, dass es den ESM-Kredit für das Gesundheitswesen ausgegeben hat? Vom Verbot der Staatsfinanzierung durch die Notenbank entsprechend Art. 123 (1) Vertrag über die Arbeitsweise der Europäischen Union (AEUV) bliebe dann nur noch wenig übrig. Es ist heute nicht möglich, vorauszusagen, wie die Europäische Zentralbank reagieren wird, sollten im Zusammenhang mit dem Corona-bedingten hohen Bedarf öffentlicher Kredite Schuldenkrisen einzelner Staaten auftreten. Es ist aber zu befürchten, dass dann gezielte Käufe der Schuldtitel dieser Staaten im Rahmen des OMT-Programms getätigt werden, obwohl diese Staaten keiner strikten makroökonomischen Stabilisierung entsprechend Art. 3, Art 12(1) ESM-Vertrag zugestimmt haben. Mit der Entscheidung, Corona-bedingte Kredite über den ESM zu finanzieren sind die Finanzminister möglicherweise einen weiteren Schritt in Richtung einer finanzpolitisch handelnden Notenbank gegangen, welche die Schwächen der Mitgliedstaaten, finanzpolitisch angemessen handeln zu können, ausbügelt.

## Corona-Bonds mit unterschiedlichem Risikopotenzial und Moral Hazard

Viele prominente deutsche Wirtschaftswissenschaftler und Politiker haben in den letzten Wochen Eurobonds vorgeschlagen (Südekum et al., 2020). Sie hoben hervor, dass die Lage anders als vor zehn Jahren beim Ausbruch der Finanzkrise ist, als ebenfalls Eurobonds vorgeschlagen wurden. Dies hat zwei Gründe. *Erstens*, die Corona-Krise ist ein katastrophaler Schock und Ausnahmefall für alle Menschen in der Europäische Union, der zur Solidarität verpflichtet. Dieses Wort erscheint siebenmal im Vertrag über die Funktionsweise der Europäischen Union. In Art. 222 (1) AEUV heißt es: „Die Union und ihre Mitgliedstaaten handeln gemeinsam im Geiste der Solidarität, wenn ein Mitgliedstaat von einem Terroranschlag, einer Naturkatastrophe oder einer vom Menschen verursachten Katastrophe betroffen ist. Die Union mobilisiert alle ihr zur Verfügung stehenden Mittel, ...“ Diese Voraussetzung liegt heute exemplarisch vor. *Zweitens* sind die zurzeit diskutierten Vorschläge zu Corona-Bonds keine vergemeinschafteten Staatsanleihen mit gesamtschuldnerischer Haftung, wie sie während der Finanzkrise vorgeschlagen und zurecht kritisiert und zurückgewiesen wurden.

Gesamtschuldnerische Anleihen, die ein hohes und unübersichtliches Risiko für den Garantiestaat auslösen, sind verfassungsrechtlich bedenklich. Sie gefährden Demokratie und Parlamentarismus, wenn internationale oder europarechtliche Verpflichtungen finanzielle Risiken entstehen lassen können, welche die Bedeutung von allgemeinen Wahlen und von Parlamentsentscheidungen aushöhlen. Das Bundesverfassungsgericht hat in seinen Entscheidungen über die Verfassungsmäßigkeit der deutschen Beteiligung am Europäischen Stabilisierungsmechanismus diesen Zusammenhang betont. Es gelangte zu dem Ergebnis, dass das höchstmögliche Risiko von 190 Mrd. Euro, das Deutschland mit seiner Quote beim ESM eingeht, bei seiner Wirtschaftskraft keine Gefahr für den demokratischen Staat birgt.[4] Es hat zudem ausgeführt, dass solche Risikoübernahmen durch den Bundestag nur dann verfassungswidrig sind, wenn sie den finanziellen Spielraum nicht nur einschränken, sondern fast völlig leerlaufen lassen können.

Die heute diskutierten und politisch vorgeschlagenen Eurobond-Modelle führen entweder zu einer begrenzten Haftung pro rata oder zu gar keiner Haftung für die Schulden anderer Staaten. Auch Mischformen, in denen die Haftungsquote eines Staats kleiner oder größer als dessen Auszahlungsquote ist, werden diskutiert. Verfassungsrechtlich am wenigsten problematisch wäre ein Corona-Bond, der den durch die Seuche entstehenden Kreditbedarf aller Staaten der Eurozone abdeckt. Haftbar wäre dann jeder Mitgliedstaat nur für jenen Betrag des Gesamtkredits, den er selbst in Anspruch nimmt. Dann können keine Rückzahlungsverpflichtungen eines Staats für einen anderen Staat entstehen. Es würde sich ein mittlerer Zinssatz zwischen den 1,6 % für italienische Staatsanleihen und -0,35 % für deutsche Staatsanleihen herausbilden, weil die Finanzmärkte das Risiko einer Staatsinsolvenz bei den einzelnen Schuldnern entsprechend deren Anteil einpreisen würden. Dieser mittlere Zinssatz liegt zurzeit bei 0,53 %.

Würden solche Corona-Anleihen im Umfang von 1 Billion Euro herausgegeben, und erhielte Deutschland eine Aus-

3 EuGH (große Kammer) C-62/14. Im Urteil zu den OMT-Käufen heißt es: „Deshalb wird durch den Umstand, dass der Ankauf von Staatsanleihen an den Sekundärmärkten unter der Bedingung, dass ein makroökonomisches Anpassungsprogramm eingehalten wird, als zur Wirtschaftspolitik gehörend angesehen, wenn dieser Ankauf vom ESM vorgenommen wird.“

4 BVerfGE 132,195 (251 ff.) und BVerfGE 135,137 (408 ff.).

zahlung von 20 % daran, so würde es einen um 0,9 % höheren Zins im Vergleich zu Bundesanleihen zahlen. Dies würde zu einer jährlichen Mehrbelastung von 1,8 Mrd. Euro führen und bei einer Corona-Anleihe mit einer Laufzeit von zehn Jahren zu einer indirekten Transferleistung an die schwächeren Länder, die nunmehr einen niedrigeren Zinssatz als für nationale Staatsanleihen zahlen würden. Nach zehn Jahren könnte die Anleihe zurückgezahlt und in nationale Staatsanleihen revolviert werden. Dies wäre ohne jegliches Bürgschaftsrisiko, führte allerdings zu einer moderaten und zeitlich begrenzten Transferleistung von 18 Mrd. Euro in zehn Jahren.

Einen solchen Vorschlag unterbreitet zurzeit Italien, wie aus einem Interview des italienischen Ministerpräsidenten Giuseppe Conte hervorgeht (Meiler, 2020). Dem Vorschlag eines so ausgestalteten Corona-Bonds wird manchmal entgegengehalten, die Corona-Anleihe sei nur der Einstieg in eine europäische Transferunion und könne nicht wieder rückgängig gemacht werden (Gerken und Van Roosebeke, 2020). Aber besondere Situationen erfordern besondere Maßnahmen, und niemand kann Deutschland zwingen, die Staatshaushalte anderer Mitgliedstaaten der Europäischen Union dauerhaft mit Eurobonds zu subventionieren. Die aktuelle Variante von Eurobonds hat nicht nur den Vorteil, verfassungsrechtlich unbedenklich zu sein. Sie belässt zudem das Haftungsrisiko ausschließlich beim Schuldner und vermindert somit den Moral Hazard.

Die Finanzierung über den ESM führt hingegen zu einer Garantiehaftung in Höhe des deutschen Kapitalanteils von 26,9 %. Das würde auch für Modelle wie jenes von Felbermayr, Hüther und anderen gelten, die Corona-Bonds nur für jene Staaten vorsehen, die Schwierigkeiten haben, die Corona-bedingte Ausweitung der Staatsschulden im nationalen Alleingang vornehmen zu können (Südekum et al., 2020). Der Vorschlag dieser Wissenschaftler hätte allerdings den Vorteil, dass wegen der Übernahme des Haftungsrisikos durch alle Staaten der Eurozone entsprechend ihrer Quote eine hohe Corona-bedingte Ausweitung der Schulden in den schwächeren Länder keinen spekulativen Angriff der Finanzmärkte auf diese Ländern auslösen könnte.

Das in diesem Zusammenhang wie ein Mantra vorgetragene Argument, eine Einheit von Haftung und Entscheidung sei notwendig, verkennt, dass einige der wichtigsten und erfolgreichsten Institutionen moderner Wirtschaftssysteme Risikostreuung, Versicherung, Risikoverlagerung und die Abwehr oder Abfederung externer Schocks ermöglichen. Ihre Einführung veränderte stets das Verhältnis von Haftung und Entscheidung und ging mit Moral Hazard einher. Ohne die Erfindung der offenen Handelsgesellschaft, die es Kaufleuten ermöglichte, Risiken zu poolen (Sinn, 1988), ohne die Erfindung der juristischen Person, die Unternehmen wirksam gegen existenzgefährdende Risiken durch die Gläubiger von Anteilseignern schützt, ohne die Kapitalgesellschaft mit begrenzter Haftung, die es durch das Trennungsprinzip ermöglicht, Teile des Privatvermögens von Gesellschaftern und Aktionären dem Gläubigerzugriff zu entziehen, gäbe es weder Kapitalmärkte noch Großunternehmen. Dass damit auch Moral-Hazard-Probleme verbunden sind, zeigt nur die Notwendigkeit auf, zwischen dem Prinzip der Risikostreuung und dem der Haftung einen Kompromiss zu finden. Erst dies ermöglicht Individuen, Unternehmen und ganzen Gesellschaften Risiken einzugehen, die andernfalls als untragbar vermieden würden.
